# Refractory Metal Intermetallic Composites, High-Entropy Alloys, and Complex Concentrated Alloys: A Route to Selecting Substrate Alloys and Bond Coat Alloys for Environmental Coatings

**DOI:** 10.3390/ma15082832

**Published:** 2022-04-12

**Authors:** Panos Tsakiropoulos

**Affiliations:** Department of Materials Science and Engineering, Sir Robert Hadfield Building, The University of Sheffield, Mappin Street, Sheffield S1 3JD, UK; p.tsakiropoulos@sheffield.ac.uk

**Keywords:** high-entropy alloys, complex concentrated alloys, refractory metal intermetallic composites, high-entropy intermetallics, complex concentrated intermetallics, alloy design

## Abstract

This paper considers metallic ultrahigh-temperature materials (UHTMs) and the alloying behaviour and properties of alloys and their phases by using maps of the parameters δ (based on atomic size), Δχ (based on electronegativity), and valence electron concentration (VEC), and discusses what connects and what differentiates material groups in the maps. The formation of high-entropy or complex concentrated intermetallics, namely 5-3 silicides, C14 Laves and A15 compounds, and bcc solid solutions and eutectics in metallic UHTMs and their co-existence with “conventional” phases is discussed. The practicality of maps for the design/selection of substrate alloys is deliberated upon. The need for environmental coatings for metallic UHTMs was considered and the design of bond coat alloys is discussed by using relevant maps.

## 1. Introduction

New materials with capabilities beyond those of Ni-based superalloys are required to meet stringent performance and environmental targets for aero engines. Industry has specified material property targets (goals) for creep, toughness, and oxidation that must be met by the new materials. The material property targets are as follows: (a) for material density 7 g/cm^3^ the creep strength should be greater than 170 MPa at a creep rate of 2 × 10^−8^ s^−1^ at 1200 °C, (b) the fracture toughness of critical components should be ≥20 MPa√m, and (c) the recession rate due to oxidation should be less than 0.25 μm/h at 1315 °C (to attain at 1315 °C the oxidation life of second-generation single-crystal Ni-based superalloys at 1150 °C) [1,2,3,4].

These property targets have stimulated research to develop metallic ultrahigh-temperature materials (UHTMs). The latter include different but related [3,4] material groups, namely refractory metal (RM) intermetallic composites (RMICs), RM high-entropy alloys (RHEAs), and RM complex concentrated alloys (RCCAs) [1,2,3,4,5]. RMICs based on Nb-Si (i.e., RM(Nb)ICs) or Mo-Si (i.e., RM(Mo)ICs) form Nb or Mo silicides and have been studied [1,3,4,5]. The toughness goal requires the UHTMs to show some degree of metallic behaviour to distinguish them from ceramic UHTMs. The latter and RM(Mo)ICs are not considered in this paper. Recent reviews on RM(Nb)ICs, RM(Mo)ICs, HEAs, RHEAs, and RCCAs can be found in [1,2,5,6]. RM(Nb)ICs were compared with RCCAs and RHEAs in [3,4].

The motivation for this paper was to draw attention to an approach to selecting (i) metallic UHTMs substrate alloys and (ii) bond coat alloys for environmental coatings for such alloys. This approach makes use of maps based on valence electron concentration, electronegativity and atomic size that link the alloying behaviour and properties of alloys and their phases and distinguish the different material groups. The paper is inspired by the research of the author and his colleagues who prompted the development of metallic UHTMs with the alloy-design methodology NICE, in which parameter maps are indispensable.

The structure of the paper is as follows. First, I briefly discuss (a) the parameters that describe alloying behaviour of metallic UHTMs and their phases, namely solid solution and intermetallics such as silicides, C14 Laves and A15 compounds and (b) the alloying elements used to date in metallic UHTMs. Then, I demonstrate that the different groups of metallic UHTMs and their phases can be shown in maps based on aforementioned parameters and briefly discuss properties of alloys and phases, in particular the bcc solid solution and tetragonal Nb_5_Si_3_ silicide. Moreover, I draw attention to the fact that in metallic UHTMs “conventional” phases can co-exist with high-entropy or complex concentrated phases, namely bcc solid solution(s), silicides, A15-Nb_3_X (X = Al, Ge, Si, Sn) compounds, C14-NbCr_2_ Laves phase, and eutectics with solid solution and tetragonal Nb_5_Si_3_. Next, I briefly deliberate on the practicality of the maps for alloy design and selection, and finally, I consider the need for environmental coatings for metallic UHTMs and discuss the design of bond coat alloys by using appropriate maps for bond coat alloys.

## 2. Parameters

Metallurgists use the enthalpy of mixing (ΔH_mix_), entropy of mixing (ΔS_mix_), atomic size (r), electronegativity (χ), or the electron-to-atom ratio to study the alloying behaviour in alloys. Likewise, the alloying behaviour of high-entropy alloys (HEAs) is studied by using analogous parameters, namely the parameters δ (based on r), Δχ (based on χ), ΔH_mix_, ΔS_mix_, Ω (=T_m_ΔS_mix/_|ΔH_mix_|), and the number of valence electrons per atom filled into the valence band (VEC) [6]. Moreover, the same parameters can describe the alloying behaviour of RM(Nb)ICs, RHEAs, and RCCAs [1,2,3,4,7], as well as of bcc solid solutions in RM(Nb)ICs [8]. In the latter materials, typical phases are bcc solid solution(s) and silicides [1,8,9,10,11,12,13], with/without other intermetallics such as A15-Nb_3_X compounds or C14-NbCr_2_ Laves phase [14] and eutectics containing the solid solution and Nb_5_Si_3_ silicide [15]. The calculation of the parameters VEC and Δχ for the said intermetallics was described in [9,14]. Note that RHEAs and RCCAs with A15-Nb_3_X compounds in their microstructure have not been studied [2].

The calculation of all the aforementioned parameters for alloys and their phases requires high-quality chemical analysis data about the actual chemical composition of alloys and their phases, meaning data that is produced using EPMA and EDS with standards [1,3,4,7,8,9,14,15]. Standardless analysis data and nominal compositions would not suffice. Unfortunately, in the large literature for metallic UHTMs high-quality chemical analysis data for alloys and their phases is rare.

The values of the aforementioned parameters for metallic UHTMs, HEAs, and amorphous alloys overlap [4,7,8]. The significance of this fact was understood in our research group and motivated the development of the new alloy design methodology, Niobium Intermetallic Composite Elaboration (NICE) [1,4], which subsequently made possible (a) the construction of maps that (i) describe the alloying behaviour of RM(Nb)ICs, RM(Nb)ICs/RCCAs (i.e., RM(Nb)ICs that are also RCCAs), and RM(Nb)ICs/RHEAs (i.e, RM(Nb)ICs that are also RHEAs) [4,7], (ii) link RM(Nb)ICs/RCCAs and RM(Nb)ICs/RHEAs with “conventional” alloys (e.g., [10,11,12,13]), and (iii) correlate these parameters with properties of alloys or their phases [1,3,4,9,14,15]; and (b) the comparison of RM(Nb)ICs, RM(Nb)ICs/RCCAs and RM(Nb)ICs/RHEAs with RCCAs and RHEAs with addition of Nb and with/without addition of Si [3,4].

Metallic UHTMs can be single-phase solid solution alloys or multiphase alloys. All RM(Nb)ICs and RM(Nb)ICs/RCCAs or RM(Nb)ICs/RHEAs are multiphase alloys. Many metallic UHTMs comply with the standard definition of HEAs or are alloys where the higher or lower concentration of elements is above or below 35 and 5 at.%, i.e., the upper and lower limits in the standard definition. These are designated respectively RHEAs (elemental concentrations (in the nominal composition) are in the range 35 to 5 at.%) and RCCAs (elemental concentrations can be >35 and <5 at.%) [2,6].

Metallic UHTMs share the same alloying additions. In the case of RM(Nb)ICs at least 23 elements have been used, namely Al, B, C, Ce, Cr, Dy, Er, Fe, Ga, Ge, Hf, Ho, In, Mo, Nb, Si, Sn, Ta, Ti, V, W, Y, and Zr [1,3]. Currently, the elements C, Ce, Dy, Er, Fe, Ga, Ho, and In are not used in RHEAs or RCCAs [2]. Some of these elements play a key role regarding the control (a) of the oxidation in the pest regime and at high temperatures. These include elements Al, B, Ce, Cr, Fe, Ge, Hf, Mo, Si, Sn, and Ti, all of which have been used only in RM(Nb)ICs and RM(Nb)ICs/RCCAs or RM(Nb)ICs/RHEAs [1,3,4,12,13] and Al, Cr, Hf, Si, and Ti only in RHEAs and RCCAs [2]; (b) of the low- and high-temperature strength and creep, for example the elements Hf, Mo, Nb, Si, Ta, Ti, W, V, and Zr (note that currently there is data for the creep only of RM(Nb)ICs and RM(Nb)ICs/RCCAs [1,3]); (c) of the fracture toughness, for example the elements Al, Cr, Hf, Mo, Si, Ti, W, and Zr [1,3,16]; (d) of vol.% of bcc solid solution in RM(Nb)ICs and RM(Nb)ICs/RCCAs or RM(Nb)ICs/RHEAs (for example the elements Al, B, Ge, Hf, Mo, Sn, Ti, Ta, and W); (e) of vol.% of A15-Nb_3_X compounds or C14-NbCr_2_ Laves phase (for example the elements Al, Cr, Ge, Mo, Si, Sn, and Ti); and (f) of the type of bcc solid solution(s) in RM(Nb)ICs and RM(Nb)ICs/RCCAs or RM(Nb)ICs/RHEAs, which can be Ti-rich or Si-free or B-free [8,12,13] (for example the elements B, Hf, Mo, Ti, W [1,3,4,8,13]). Note that (d), (e), and (f) are key to meeting the three property goals that were given in the introduction. Finally, alloying additions in metallic UHTMs can be grouped together taking into account atomic size, electronegativity, elastic moduli, and diffusivity [1,3,4,8]. Boron always belongs in distinct different groups [8].

## 3. Alloy Maps

**Figure 1:**
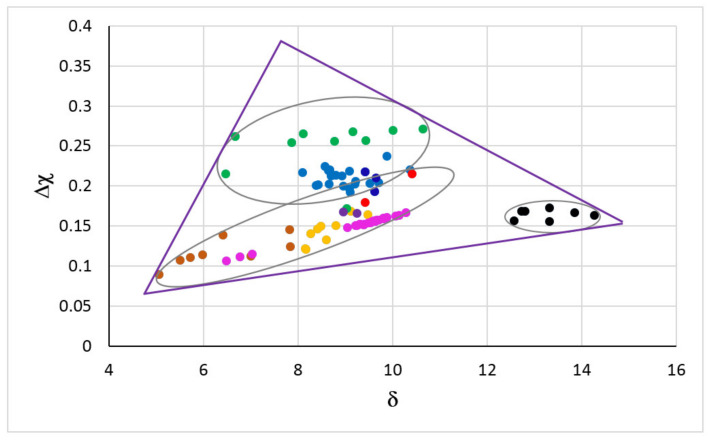
Δχ versus δ map of RM(Nb)ICs, RM(Nb)ICs/RCCAs, RM(Nb)ICs/RHEAs and RHEAs or RCCAs with addition of Nb and Si. RM(Nb)ICs: Orange circles for alloys of B-free Nb-SM-Met-TM-RM systems with Nb_ss_ and Nb_5_Si_3_ with/without Laves phase; blue circles for alloys of B-free Nb-Si-TM-RM systems with Nb_ss_ and Nb_5_Si_3_; black circles for alloys of Nb-SM-B-TM-RM systems with Nb_ss_ and 5-3 silicide alloyed with boron [7]. SM = simple metal and Met = metalloid element (Al, B, Ge, Si, Sn). TM = transition metal (Cr, Hf, Ti); RM=Mo, Ta, W. RHEAs and RCCAs; green circles for alloys with bcc solid solution plus M_5_Si_3_ silicide(s) [2]; red circles for alloys with bcc solid solution plus M_5_Si_3_ silicide(s) and Laves phase(s) [2]; darker orange circles for alloys with bcc solid solution plus Laves phase without M_5_Si_3_ silicide [17] (M = TM, RM). RM(Nb)ICs/RCCAs; purple circles for the alloys OHS1 [18] and ZF9 [19] with Nb_ss_ and Nb_5_Si_3_; dark blue circles for the alloys JZ3+, JZ4 and JZ5 with Nb_ss_ and Nb_5_Si_3_ [20,21]. RM(Nb)ICs/RHEAs; pink circles for the alloys MG5, MG6 and MG7 with Nb_ss_ and Nb_5_Si_3_ and other intermetallics [11,22]. For triangle see text. For the nominal compositions of the alloys see the Appendix A.

The map for metallic UHTMs with Nb and Si additions is shown in the Figure 1. The map is based on the parameters Δχ and δ, and is the “master” map of RM(Nb)ICs, RM(Nb)ICs/RCCAs, RM(Nb)ICs/RHEAs, and RHEAs or RCCAs with Nb and Si additions [3]. In this figure the RM(Nb)ICs shown with orange, blue and black circles correspond to the alloys respectively of the groups A, B and C in the Figure 1 in [7] and Figure 19 in [3]. The triangle delineates the area occupied by metallic UHTMs in the master map [3]. Note the distinct different area that is occupied by B containing metallic UHTMs.

**Figure 2:**
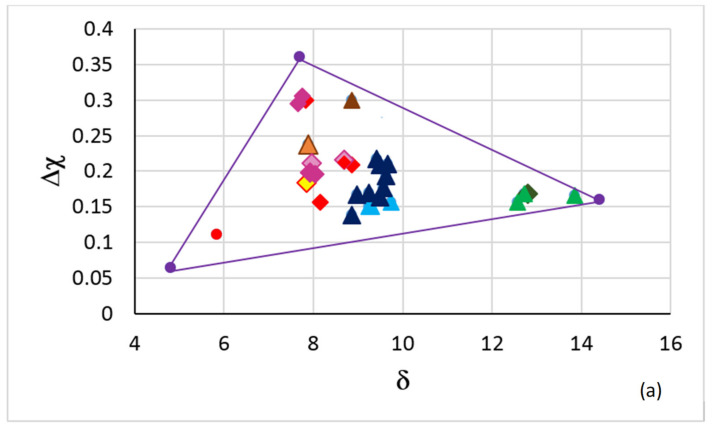

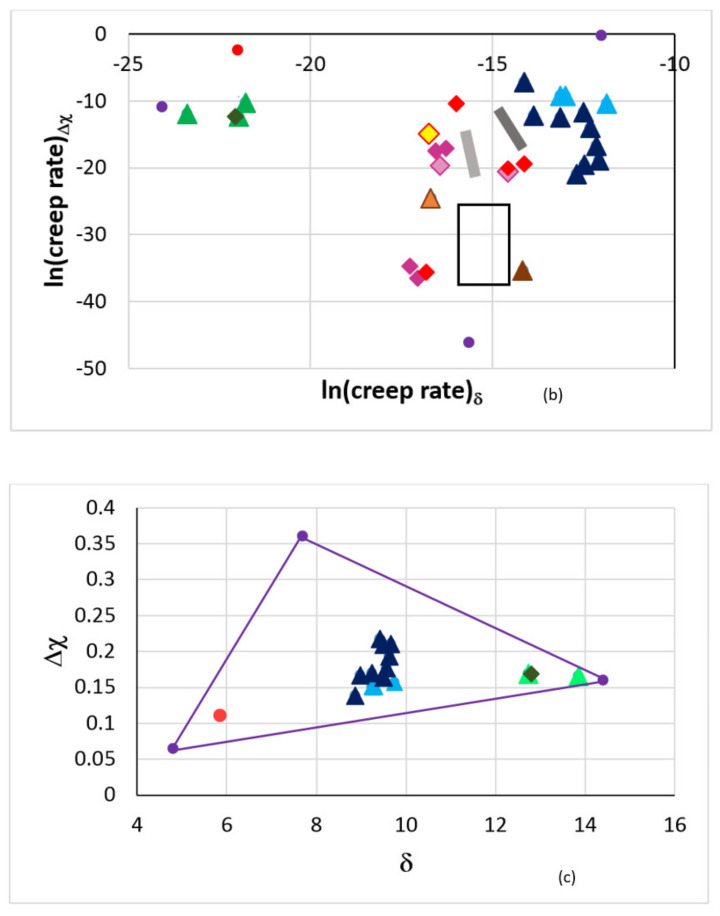

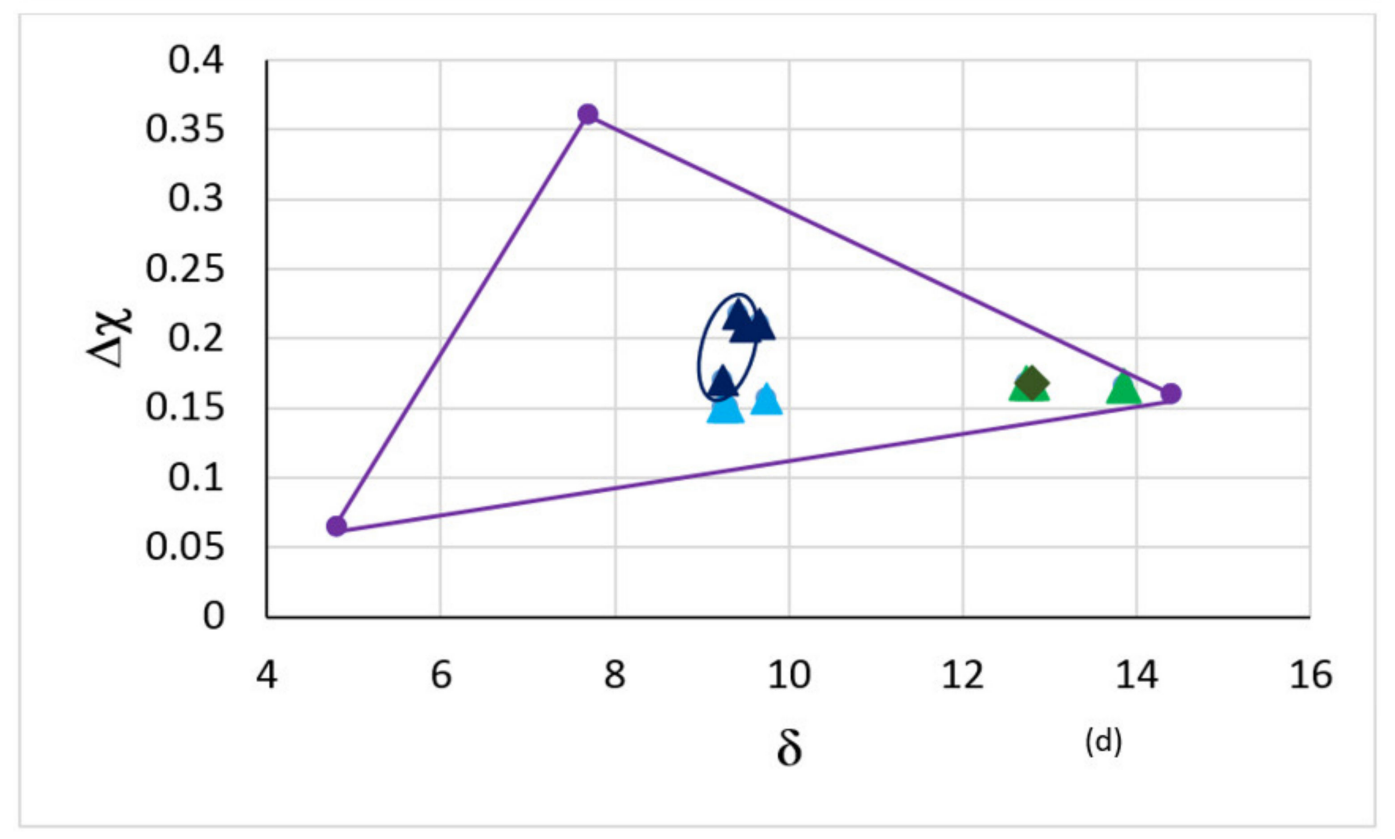
(**a**) Δχ versus δ map of selected RM(Nb)ICs, RM(Nb)ICs/RCCAs and RM(Nb)ICs/RHEAs (see text). (**b**) Map of creep rates at 1200 °C and 170 MPa (creep goal conditions) of the alloys included in (**a**). (**c**) Δχ versus δ map of alloys in (**a**) that after 100 h at T ≤ 800 °C did not pest. (**d**) Δχ versus δ map of alloys in (**a**) that had exceptional oxidation after 100 h at 800 °C and for 100 h at 1200 °C, meaning alloys that did not pest and whose scales that formed at 800 and 1200 °C did not spall off. In (**a**–**d**) circle and diamonds are for RM(Nb)ICs and triangles for RM(Nb)ICs/RCCAs or RHEAs. In (**a**,**c**,**d**) the purple triangle corresponds to the triangle in the Δχ versus δ master map in the Figure 1. See Appendix A for the nominal alloy compositions. RM(Nb)ICs: NV1, Ti-free alloys YG4 to YG6, YG8 and XX1, and Ti-containing alloys PT1, PT2 to PT4, and XX3, XX4, and XX6. Boron-free RM(Nb)ICs/RCCAs: JG6, OHS1, ZF9, EZ8, JZ4, JZ5, NT1.1, ZX8, XX2 and XX5. Boron- containing RM(Nb)ICs/RCCAs: TT5, TT6, TT7. Boron-containing RM(Nb)IC: TT8. RM(Nb)ICs/RHEAs: MG5, MG6 and MG7. Red circle for the alloy NV1. Red diamonds for the creep- resistant alloys YG4 to YG6, YG8 and XX1. Pink diamonds for creep-resistant alloys PT1, PT2, PT3, PT4, XX3, XX4, XX6 (for light pink diamonds and yellow diamond see text). Dark green diamond for the alloy TT8. Dark blue triangles for B free RM(Nb)ICs/RCCAs. Green triangles for the alloys TT5, TT6, and TT7. Brown triangles for the creep-resistant alloys XX2 and XX5 (for the light brown triangle see text). Light blue triangles for the alloys MG5, MG6, and MG7. In (**c**) the alloys are NV1, JG6, OHS1, ZF9, EZ8, JZ4, JZ5, NT1.1, TT6, TT7, TT8, ZX8, and MG5, MG6, and MG7. In (**d**) the alloys are OHS1, JZ4, JZ5, NT1.1, TT6, TT7, TT8 and MG5, MG6, and MG7 and the ellipse contains the alloys OHS1, JZ4, JZ5 and NT1.1 with simultaneous addition of Ge and Sn.

Figure 2a shows the triangle of the map in Figure 1 with selected creep-resistant or oxidation-resistant RM(Nb)ICs, RM(Nb)ICs/RCCAs, and RM(Nb)ICs/RHEAs. Note (i) that RHEAs and RCCAs with Nb and Si addition are not included in Figure 2a because there is no creep data for these metallic UHTMs [2]; (ii) that in the corner occupied by B containing RM(Nb)ICs in Figure 1 there are B containing RM(Nb)ICs/RCCAs in Figure 2a; (iii) that the RM(Nb)IC alloy NV1 (red circle, for nominal composition see Appendix A), which essentially is a solid solution alloy (80 vol.% Nb_ss_, 20% Nb_5_Si_3_), is in the area in Figure 1 that is occupied by RHEAs and RCCAs with bcc solid solution plus Laves phase but without M_5_Si_3_ silicide(s); (iv) that in the other corner near the area in Figure 1 that is occupied by RHEAs and RCCAs with bcc solid solution plus M_5_Si_3_ silicide(s) (green circles in Figure 1) and above it there are creep-resistant RM(Nb)ICs and RM(Nb)ICs/RCCAs in Figure 2a; and (v) that creep-resistant RM(Nb)ICs in Figure 2a are also in the area in Figure 1 that is below the green circles and above Δχ about 0.15, but not RM(Nb)ICs/RCCAs.

In NICE, there are relationships that link the creep of RM(Nb)ICs and the parameters δ, Δχ, and VEC. These relationships allow one to calculate (predict) the average creep rate <έ> due to intrinsic resistances to dislocation mobility, not extrinsic ones (i.e., grain boundaries, anti-phase boundaries, twins, precipitates etc. [1,3]). The contribution to creep of each of the aforementioned parameters, namely έ_δ_, έ_Δ__χ_ or έ_VEC_, to <έ> is constrained by alloying additions, type and vol.% of Nb_ss_ and silicide(s) and alloying element ratios [1,3,4]. Alloying additions decrease or increase the creep rate; for example see the Figure 22 in [4] for the effect of Al, Mo, Si or Ti additions in RM(Nb)ICs on lnέ_VEC_ for the creep goal condition (1200 °C, 170 MPa). (Processing can change the properties of RM(Nb)ICs and RM(Nb)ICs/RCCAs [3]. NICE does not consider processing and microstructure architecture (e.g., spatial distribution of phases, their shape, and size) [1]).

The creep rates in Figure 2b were calculated by using NICE for the creep goal condition (1200 °C, 170 MPa). The purple circles in Figure 2b correspond to the corners of the purple triangles in the Figure 1 and Figure 2a,c,d. The creep rates corresponding to the purple points were calculated assuming that the creep rate equations in NICE are applicable to very low and very high parameter values. Furthermore, the calculations for the B-containing alloys have not taken into account the presence of the hexagonal D8_8_ silicide in their microstructure [12,13], and therefore it is highly likely that for these alloys the έ_δ_ and έ_Δ__χ_, respectively, was overestimated (should be higher) and underestimated (should be lower).

How does experimental data for creep compare with that calculated with NICE for RM(Nb)ICs and RM(Nb)Ics/RCCAs? Data about the creep of RM(Nb)Ics is given in [3] and data about the creep of the phases that can form in RM(Nb)Ics is given in [1,9]. The measured minimum creep rates in compression for the creep goal condition (1200 °C, 170 Mpa) for the Ti and Hf containing RM(Nb)Ics alloys PT1 and PT3 (both with Ti/Si = 0.55 [3], see the Appendix A for the nominal compositions), shown by the left and right light pink diamonds in Figure 2b, were 5.8 × 10^−8^ s^−1^ (−16.66) and 5.1 × 10^−7^ s^−1^ (−14.49), respectively, where in parentheses is given the lnέ. For the same alloys, the creep rates that were calculated by using NICE and the relationships based on the parameters VEC, Δχ, and δ were, respectively 1 × 10^−7^ s^−1^ (−16.12), 2.5 × 10^−9^ s^−1^ (−19.8), 7.2 × 10^−8^ s^−1^ (−16.44) for PT1, and 4.5 × 10^−7^ s^−1^ (−14.6), 1.1 × 10^−9^ s^−1^ (−20.66), and 4.7 × 10^−7^ s^−1^ (−14.57) for PT3. Note (i) that the alloy PT3 had higher concentration of Hf and higher vol.% Nb_ss_; and (ii) that for both alloys the έ_δ_ and έ_VEC_ are close to έ_experiment_.

For the Ti-containing but Hf-free RM(Nb)IC/RCCA alloy XX5 (Ti/Si = 1.2 [3], light brown triangle in Figure 2b), the measured compressive creep rate at 1397 °C and 200 Mpa was about 10^−6^ s^−1^ [3,23], whereas the calculated creep rate for 200 Mpa and 1200 °C, i.e., for 200° lower than the experimental measurements, was 1.8 × 10^−7^ s^−1^, 3.6 × 10^−10^ s^−1^ and 1 × 10^−7^ s^−1^ based on the parameters VEC, Δχ, and δ, respectively. For the RM(Nb)IC alloy NV1 (Ti/Si = 4.6, with 80 vol.% Nb_ss_) the calculated έ_Δ__χ_ was about eight orders higher than the έ_δ_ that was about five orders lower than the έ_VEC_ (1 × 10^−5^ s^−1^) at 1200 °C and 170 Mpa. The creep of NV1, owing to its very high vol.% of solid solution, was as expected very poor at the creep goal conditions.

For the Ti- and Hf-free RM(Nb)IC alloy YG6 [24] (yellow diamond in Figure 2b, see Appendix A for the nominal composition), the measured creep rate in compression for the creep goal condition was 6.3 × 10^−8^ s^−1^ (−16.59) compared with έ based on the parameters VEC, Δχ, and δ that was 2 × 10^−8^ s^−1^ (−17.73), 2.7 × 10^−7^ s^−1^ (−15.12), and 5.3 × 10^−8^ s^−1^ (−16.75), respectively. For this alloy, the creep rate έ_δ_ is close to the experimental έ. Note (i) that in the case of the alloy YG6 the έ_Δ__χ_ is about one order higher than έ_VEC_ or έ_δ_, whereas it is about one or two orders lower than έ_VEC_ or έ_δ_ in the case of the alloys PT1 and PT3; (ii) that the vol.% Nb_ss_ was lower in YG6 compared with PT1 and PT3; and (iii) that the Si free Nb_ss_ was stable in the alloys YG6, PT1, PT3 and XX5 but not in the alloy NV1.

The alloys in Figure 2a that had very good oxidation after 100 h at a pest temperature (T ≤ 800 °C) are shown in the Δχ versus δ map in the Figure 2c. Very good oxidation at a pest temperature means the alloys did not form the “Maltese cross”, did not pest and their scales did not spall off. Figure 2c shows narrow ranges of values of the parameters δ and Δχ for very good oxidation for Ti-containing RM(Nb)Ics, RM(Nb)Ics/RCCAs, and RM(Nb)Ics/RHEAs. The alloys in Figure 2a that had exceptional oxidation behaviour for 100 h at 800 °C and for 100 h at 1200 °C (meaning the alloys did not form the Maltese cross) did not pest and their scales that formed at the pest temperature and at 1200 °C did not spall off, are shown in the Δχ versus δ map in the Figure 2d. The alloys in the ellipse in Figure 2d are the RM(Nb)Ics/RCCAs OHS1, JZ4, JZ5, and NT1.1 (see Appendix A for the nominal compositions). In all these alloys, Ge and Sn were present simultaneously. Note (a) that the oxidation-resistant alloys had 7.3 × 10^−7^ s^−1^ < έ_δ_ < 7.1 × 10^−6^ s^−1^ and 7.8 × 10^−10^ s^−1^ < έ_Δ__χ_ < 7.8 × 10^−4^ s^−1^ (Figure 2b); and (b) the presence of the alloy NV1 in the Figure 2c but not in the Figure 2d (meaning the alloy NV1 had very good oxidation at pest temperatures owing to the chemical composition of the Nb_ss_, even though the vol.% of Nb_ss_ was very high, but its oxidation at 1200 °C was poor because of the high vol.% of Nbss).

## 4. Phases’ Master Map and Properties

**Figure 3:**
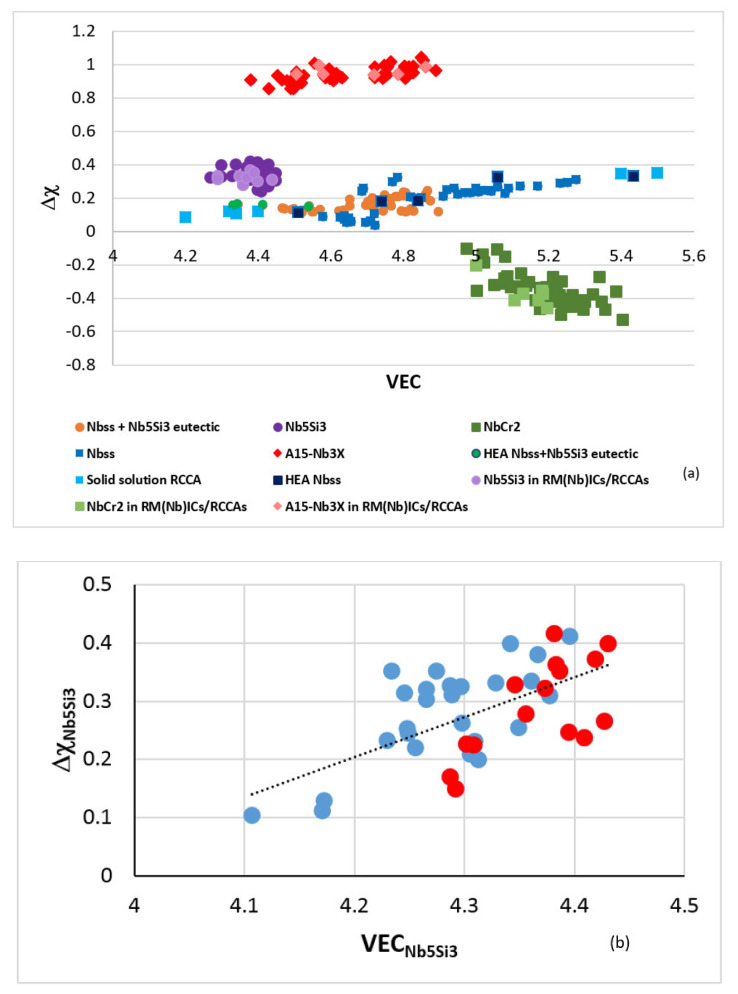

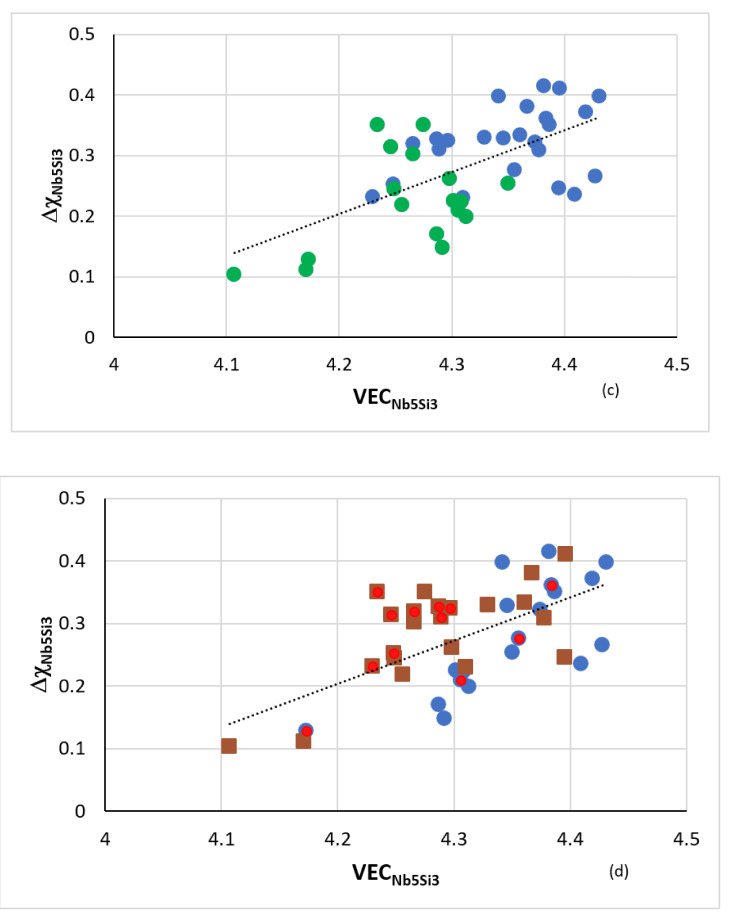

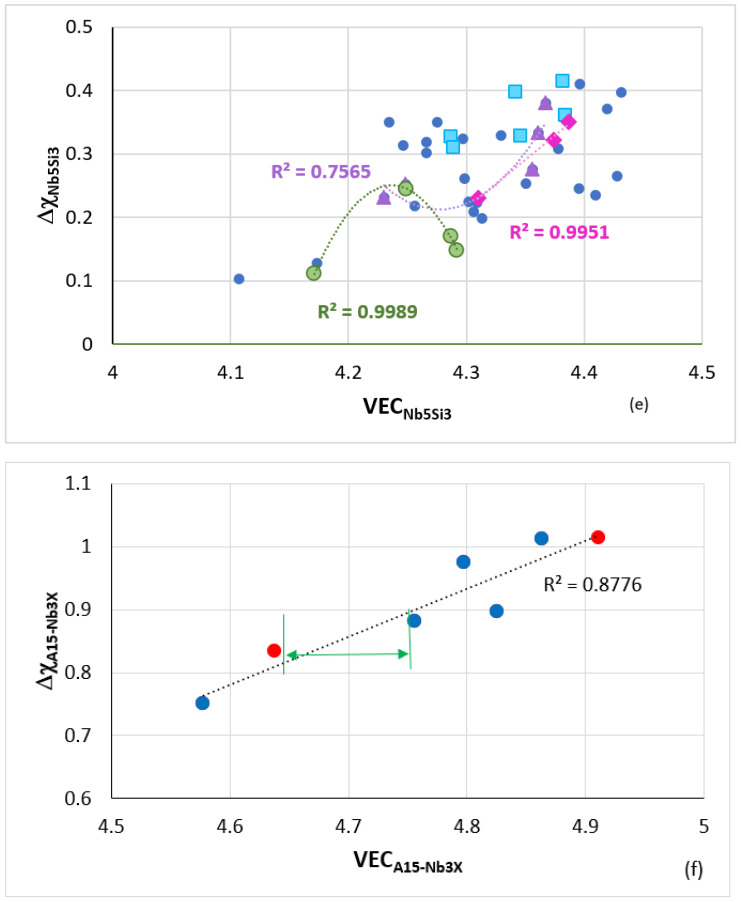
(**a**) Δχ versus VEC map of phases in RM(Nb)ICs, RM(Nb)ICs/RCCAs, and single-phase RCCAs. The single-phase RCCAs contain Nb but not Si. The data is for bcc Nb_ss_ [8], Nb_5_Si_3_ [9], C14-NbCr_2_ Laves [14], eutectics of bcc Nb_ss_ + βNb_5_Si_3_ [15], A15-Nb_3_X [14], the bcc solid solution RCCAs studied by Senkov et al. [2], the HEA bcc Nb_ss_, and the HEA bcc Nb_ss_ + βNb_5_Si_3_ eutectics in RM(Nb)ICs [1,15]. The RCCAs with high VEC values are the NbMoTaW and NbMoTaWV alloys [2], the RCCAs with lower VEC values have Al, Nb and Ti additions with/without Hf [2]. (**b**–**e**) Δχ versus VEC maps of alloyed tetragonal Nb_5_Si_3_ in RM(Nb)ICs and RM(Nb)ICs/RCCAs. (**b**) shows high-entropy silicides (blue circles) and complex concentrated silicides (red circles). In (**c**), the B-alloyed T2-Nb_5_(Si,B)_3_ silicides are shown with green circles. In (**d**) the Ti-rich Nb_5_Si_3_ is shown with brown squares and the Hf-rich Nb_5_Si_3_ with red circles (note that the Nb_5_Si_3_ can be Ti-rich or Hf-rich or Ti and Hf-rich (e.g., see [9,19,20,21,25,26]), the latter is the case when the metallic UHTM contains both elements). In (**b**–**d**) the dashed lines are drawn to help the eye see the trend in the data. In (**e**), the tetragonal Nb_5_Si_3_ that is alloyed with B, Ge, Sn or Ge + Sn is shown with light green circles, light blue squares, purple triangles, and pink diamonds, respectively. The data for the alloyed tetragonal Nb_5_Si_3_ is for the alloys EZ8, JG2, JN1, KZ6, OHS1, TT4, TT5, TT6, TT7, TT8, ZF6, ZF9, and ZX8 (see Appendix A for the nominal compositions). (**f**) Δχ versus VEC map of alloyed A15-Nb_3_X in RM(Nb)ICs, and RM(Nb)ICs/RCCAs. The data is for the A15 compounds in the Table 1. The blue circles indicate A15-Nb_3_X high entropy compounds. The gap in VEC values is shown with the green double arrow.

Similarly to the “master” map of metallic UHTMs with Nb and Si addition shown in Figure 1, there is a master map for the key phases in these alloys, namely the bcc solid solution, tetragonal Nb_5_Si_3_ silicide, A15-Nb_3_X compounds and C14-NbCr_2_ Laves phase (no RCCAs or RHEAs with A15-Nb_3_X compounds have been studied [2]). The master map of phases is the Δχ versus VEC map shown in Figure 3a and displays the phases in RM(Nb)Ics and RM(Nb)Ics/RCCAs, the HEA Nb_ss_ and HEA Nb_ss_+Nb_5_Si_3_ eutectics in RM(Nb)Ics and single-phase solid solution RCCAs studied by Senkov et al. [2]. Note that data about the phases in multiphase RHEAs and RCCAs with Nb and Si addition is not included in this map because there is no data about the actual chemical composition of phases in the said materials [2]; such data is indispensable for the calculation of the parameters, as discussed in Section 2.

In the Figure 3a note (a) that the data for the bcc solid solution and for Nb_ss_+βNb_5_Si_3_ eutectic includes solid solutions and eutectics in RM(Nb)Ics whose actual chemical composition satisfies the “standard definition” of HEAs [1,15] but excludes the data for bcc Nb_ss_ alloyed with B; and (b) that the data for Nb_5_Si_3_ excludes that for the tetragonal T2-Nb_5_(Si,B)_3_ silicide. Data for the B alloyed Nb_ss_ and T2-Nb_5_Si_3_ in the Δχ versus VEC master map of metallic UHTMs can be found in Figure 10a in [4] and in Figure 5a in [1]. Be aware that in Figure 5a in [1], the labels for A15-Nb_3_X and C14-NbCr_2_ Laves were swapped by mistake. Also, in Figure 3a, notice (c) the gap in VEC values for the A15-Nb_3_X compounds [4,14], and (d) that this master map cannot show the gap in Δχ values of the bcc Nb_ss_ [1,3,4,8]. Additionally, take notice of the facts (e) that the single- phase bcc solid solution RCCAs studied by Senkov et al. [2] contained Nb but not Si; (f) that the single-phase bcc solid solution RCCAs with low VEC values (i.e., the RCCAs with addition of Al, Hf, or Ti to improve oxidation resistance) are located on the left of the area occupied by bcc Nb_ss_ and B alloyed bcc Nb_ss_; and (g) that the improved oxidation behaviour of these RCCAs is in agreement with the prediction of NICE according to which the oxidation of metallic UHTMs with Nb and Si addition improves as the alloy parameter VEC decreases [1].

The phases that are observed in the microstructures of RM(Nb)Ics and RM(Nb)Ics/RCCAs or RM(Nb)Ics/RHEAs often satisfy the standard definition of HEAs or complex concentrated alloys (CCAs) [1,3,8,9]. Table 1 gives examples of bcc solid solutions (Nb_ss_), tetragonal Nb_5_Si_3_ silicides, eutectics with Nb_ss_ and tetragonal Nb_5_Si_3_, A15-Nb_3_X (X = Al, Ge, Si, Sn) compounds, and C14-NbCr_2_ Laves phase that are high-entropy or complex concentrated phases (meaning high entropy silicides or complex concentrated silicides etc.) in RM(Nb)Ics and RM(Nb)Ics/RCCAs. It should be noted that the hexagonal B alloyed 5-3 silicide (D8_8_ silicide) also can be complex concentrated silicide; for example see the data for D8_8_ in the alloy TT5-AC and TT5-HT in [13]. Such phases can co-exist with conventional phases in these metallic UHTMs.

The data in Table 1 shows that in some metallic UHTMs more than one high-entropy or complex concentrated phase can be present in the same alloy condition. For example, in Table 1 see data for the RM(Nb)IC alloy NV1-AC (Nb_ss_, Nb_5_Si_3_ and eutectic), the RM(Nb)IC/RCCA alloy JZ5-HT (Nb_ss_, Nb_5_Si_3_ and A15- Nb_3_X), the RM(Nb)IC/RCCA alloys EZ8-AC, OHS1, JZ3^+^-HT, and TT5-AC (Nb_ss_ and Nb_5_Si_3_). Also notice that in other metallic UHTMs in Table 1 one phase can be present (for example the RM(Nb)IC alloy ZX4-AC (C14-NbCr_2_ Laves phase) or the RM(Nb)IC/RCCA alloy ZF9 (Nb_5_Si_3_)), and that in the same alloy partitioning of solutes can result to the formation of more than one high-entropy or complex concentrated phases in the same condition (for example the RM(Nb)Ics/RCCA alloy JZ5 (data for the A15-Nb_3_X)).

Remarkably, during the solidification and/or heat treatment of RM(Nb)Ics, RM(Nb)Ics/RCCAs or RM(Nb)Ics/RHEAs specific phase(s) can form that can be high-entropy or complex concentrated phases (see Table 1). These phases are formed in-situ owing to solidification conditions and/or solute partitioning (e.g., see [11]) and co-exist with “conventional” phases (e.g., the RM(Nb)IC alloys KZ6-AC, JG2-HT and ZF6-HT where complex concentrated Nb_5_Si_3_ silicides coexisted with “conventional” Nb_ss_ or the RM(Nb)IC alloy ZX8-AC where the C14-NbCr_2_ complex concentrated Laves phase co-existed with “conventional” A15-Nb_3_X and βNb_5_Si_3_, whereas in the heat-treated condition, the αNb_5_Si_3_ complex concentrated silicide co-existed with “conventional” Nb_ss_, A15-Nb_3_X and C14-NbCr_2_ Laves phase (Table 1)). Owing to the lack of high-quality chemical analysis data for the phases in the microstructures of multiphase RCCAs and RHEAs [2], it is not possible to ascertain whether such phases (meaning high-entropy and/or complex concentrated phases) co-exist with conventional phases in these metallic UHTMs. Furthermore, RM(Nb)Ics/HEAs can form layered microstructures during solidification wherein a layer with composition corresponding to a conventional alloy forms next to layer of composition corresponding to an HEA alloy [11].

The Δχ versus VEC map of alloyed tetragonal high-entropy or complex concentrated Nb_5_Si_3_ silicides in RM(Nb)ICs, RM(Nb)ICs/RCCAs or RM(Nb)ICs/RHEAs is shown in the Figure 3b–e, where the same alloyed silicides are shown in all four parts. The parameter Δχ as VEC increases (see dashed line in Figure 3b). The separate areas occupied by high-entropy silicides and complex concentrated silicides are shown in the Figure 3b. The alloyed tetragonal T2-Nb_5_(Si,B)_3_ is a mainly high-entropy silicide (Figure 3c). Ti- or Hf-rich Nb_5_Si_3_ can be high-entropy or complex concentrated silicide (Figure 3d). Sn-alloyed T2-Nb_5_(Si,B)_3_ and Nb_5_Si_3_ is a high-entropy silicide, and Ge- or Ge+Sn-alloyed Nb_5_Si_3_ is high-entropy or complex concentrated silicide (Figure 3e).

The Δχ versus VEC map of alloyed tetragonal high-entropy or complex concentrated A15-Nb_3_X (X = Al, Ge, Si, Sn) compounds in RM(Nb)ICs, RM(Nb)ICs/RCCAs, or RM(Nb)ICs/RHEAs is shown in the Figure 3f. The parameter Δχ increases as VEC increases. There are high-entropy and complex concentrated A15 compounds on either side of the gap in VEC values, which is the same gap as that shown in the Figure 3a, and in [14].

**Figure 4:**
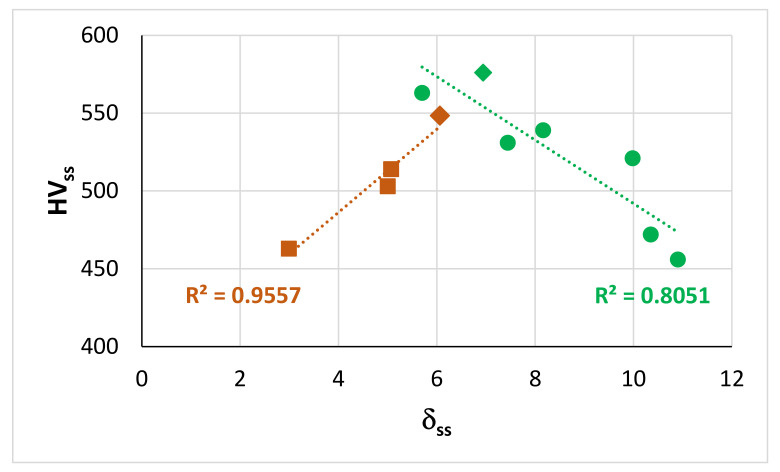
Relationships between HV_ss_ versus δ_ss_ for as cast Nb-24Ti-18Si-based alloys (KZ series alloys (see Appendix A, brown data) [25,26] and for as cast Nb-24Ti-18Si-based alloys with B and RM (=Mo,Ta) addition (green data) [12,13]. The diamond data points correspond to alloys with Ta addition. For the data indicated with squares and diamonds R^2^ = 0.970.

**Figure 5:**
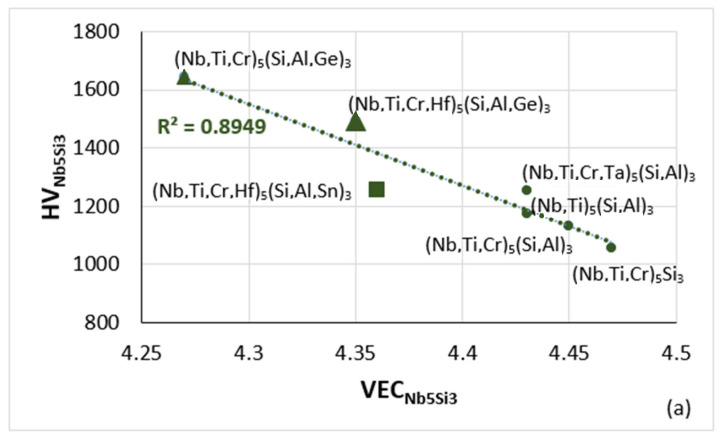

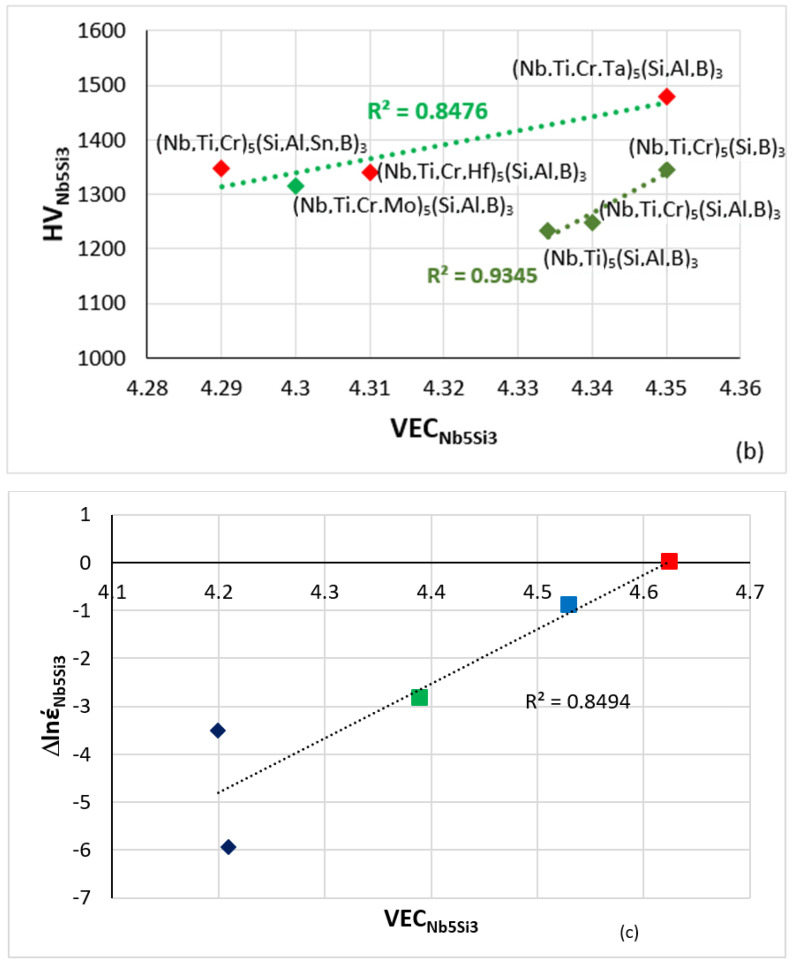
(**a**,**b**) Vickers hardness of alloyed Nb_5_Si_3_ versus VEC. (**a**) Silicide in boron-free RM(Nb)ICs, triangles and square in RM(Nb)ICs with Ge or Sn addition, respectively (**b**) Silicide in boron containing RM(Nb)ICs (diamonds), of which those that also are RM(Nb)ICs/RCCAs are shown in red. (**c**) Change of creep rate (Δlnέ) versus VEC at 1200 °C and 150 MPa with reference the binary Nb_5_Si_3_, data from [1,9]. Colours in (**c**): tetragonal unalloyed Nb_5_Si_3_ (red square), tetragonal (Nb,Ti)_5_Si_3_ (blue square), tetragonal (Nb,Ti,Cr,Hf)_5_(Si,Al,B)_3_ (green square) and hexagonal (Nb,Ti)_5_Si_3_ and (Nb,Ti,Hf)_5_Si_3_ (blue diamonds). The more negative Δlnέ is, the higher is the creep rate compared with αNb_5_Si_3_ at the said conditions.

**Figure 6:**
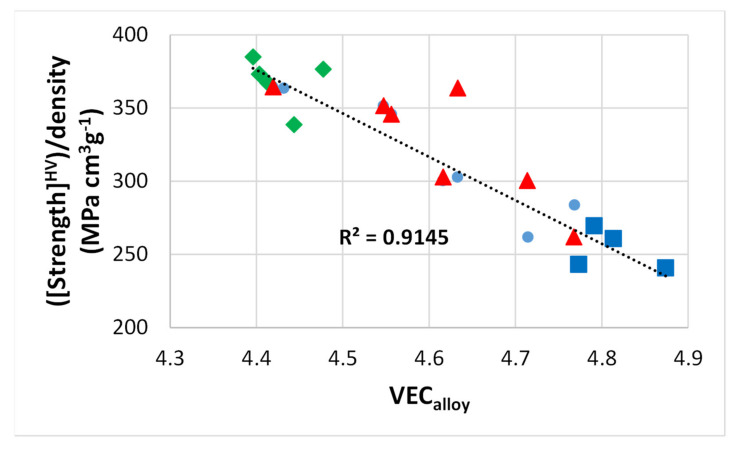
RT-specific strength (calculated by using hardness data and density of as cast alloys) versus VEC_alloy_. Green data for alloys with B [12,13,28], blue data for alloys without Ti [3], red data for Nb-24Ti-18Si KZ series alloys (see Appendix A for the nominal alloy compositions) [25,26].

As discussed here and in [9], the parameters Δχ and VEC of alloyed Nb_5_Si_3_ silicides in RM(Nb)ICs, RM(Nb)ICs/RCCAs or RM(Nb)ICs/RHEAs change upon alloying. Maps based on these parameters show the “direction of change”; for example see Figures 5 to 11 in [9] and the Figure 6 in [4]. The parameters δ, Δχ, and VEC correlate with properties of alloys and their phases; see Figure 4, Figure 5 and Figure 6 and [1,3,4]. For example, Figure 4 and Figure 5a,b show that the correlation of the hardness of the alloyed bcc solid solution with its parameter δ (Figure 4) and the hardness of the alloyed tetragonal Nb_5_Si_3_ silicide with its parameter VEC (Figure 5a,b) strongly depends on the presence of B in the RM(Nb)ICs or RM(Nb)ICs/RCCAs, whereas Figure 5c shows that the creep of Nb_5_Si_3_ deteriorates upon alloying. Furthermore, the parameters δ, Δχ and VEC of phases correlate with those of alloys; see the VEC_alloy_ versus VEC_ss_, the Δχ_alloy_ versus Δχ_ss_ and VEC_alloy_ versus VEC_Nb5Si3_ correlations in Figure 6 in [3], the VEC_alloy_ versus Δχ_Nb5Si3_ correlation in Figure 7 in [4], and the Δχ_eutectic_ versus Δχ_alloy_ correlation in Figure 17c in [4]. Even though properties of the solid solution and Nb_5_Si_3_ silicide depend on the presence of B in the alloy (Figure 4 and Figure 5), the room temperature-specific strength of RM(Nb)ICs and RM(Nb)ICs/RCCAs calculated from hardness decreases with increasing VEC_alloy_, and the correlation is the same for B-free and B-containing alloys and B-containing RM(Nb)ICs and RM(Nb)ICs/RCCAs have very high specific strengths owing to their low densities, as shown in Figure 6 [12,13].

**Figure 7:**
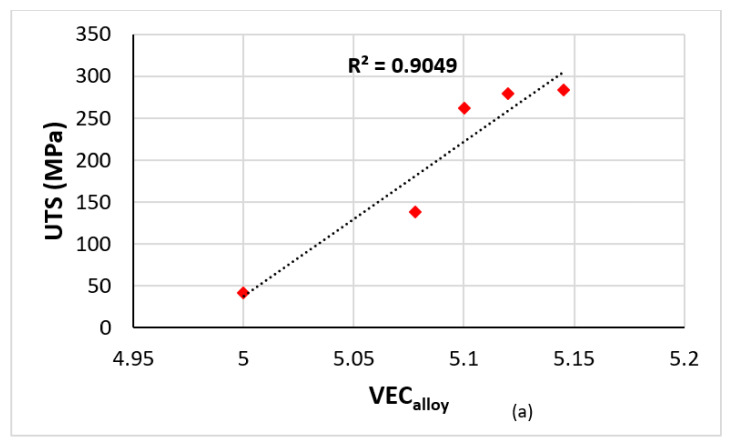

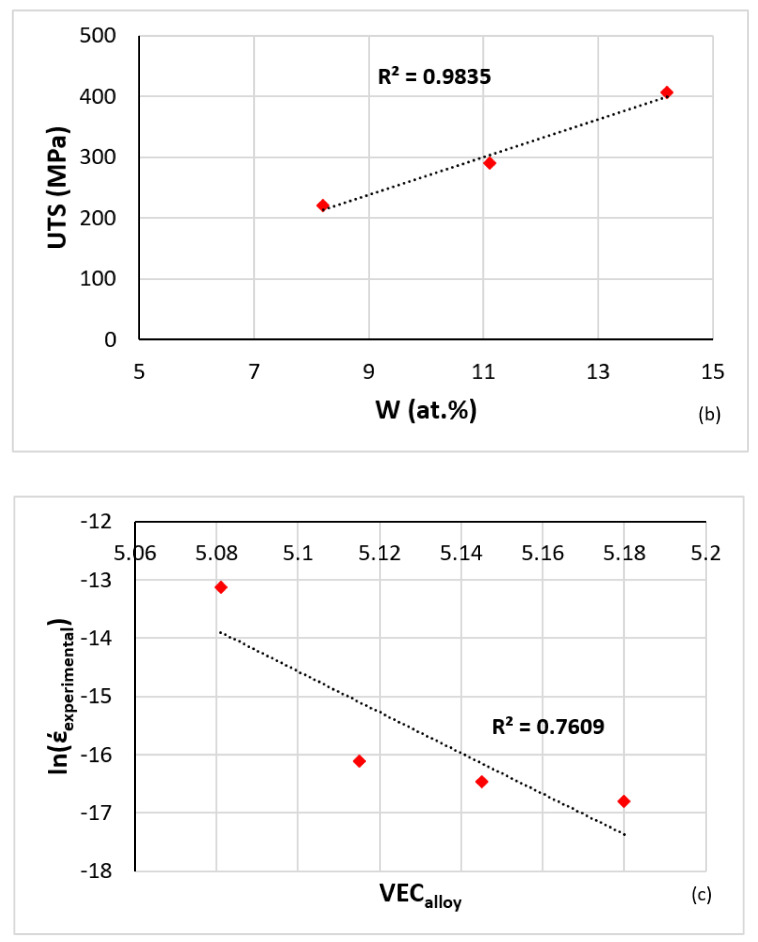

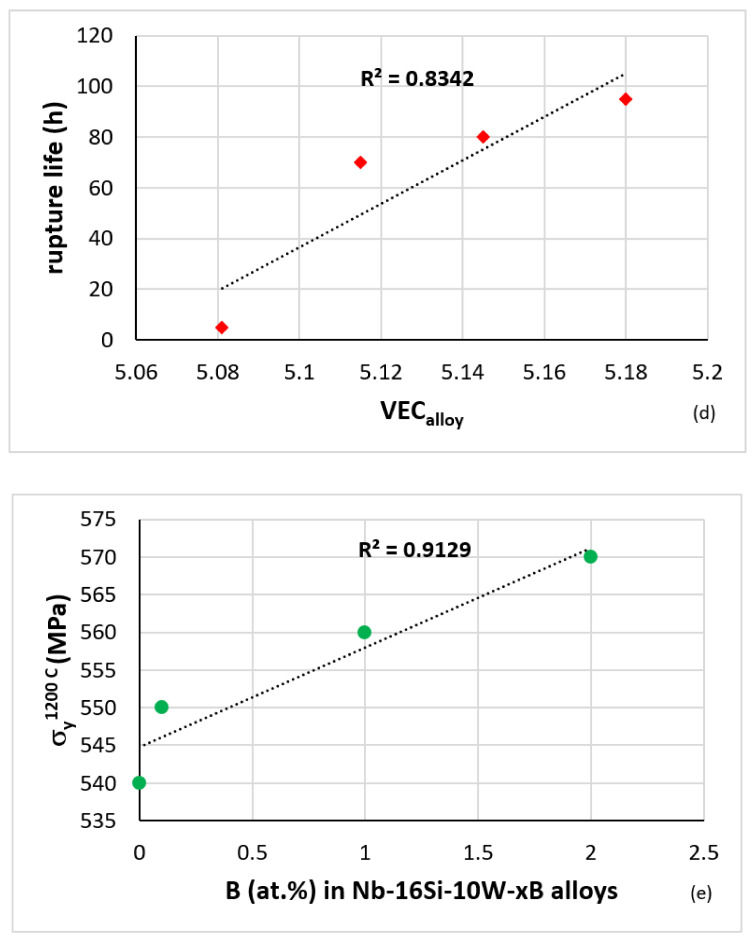

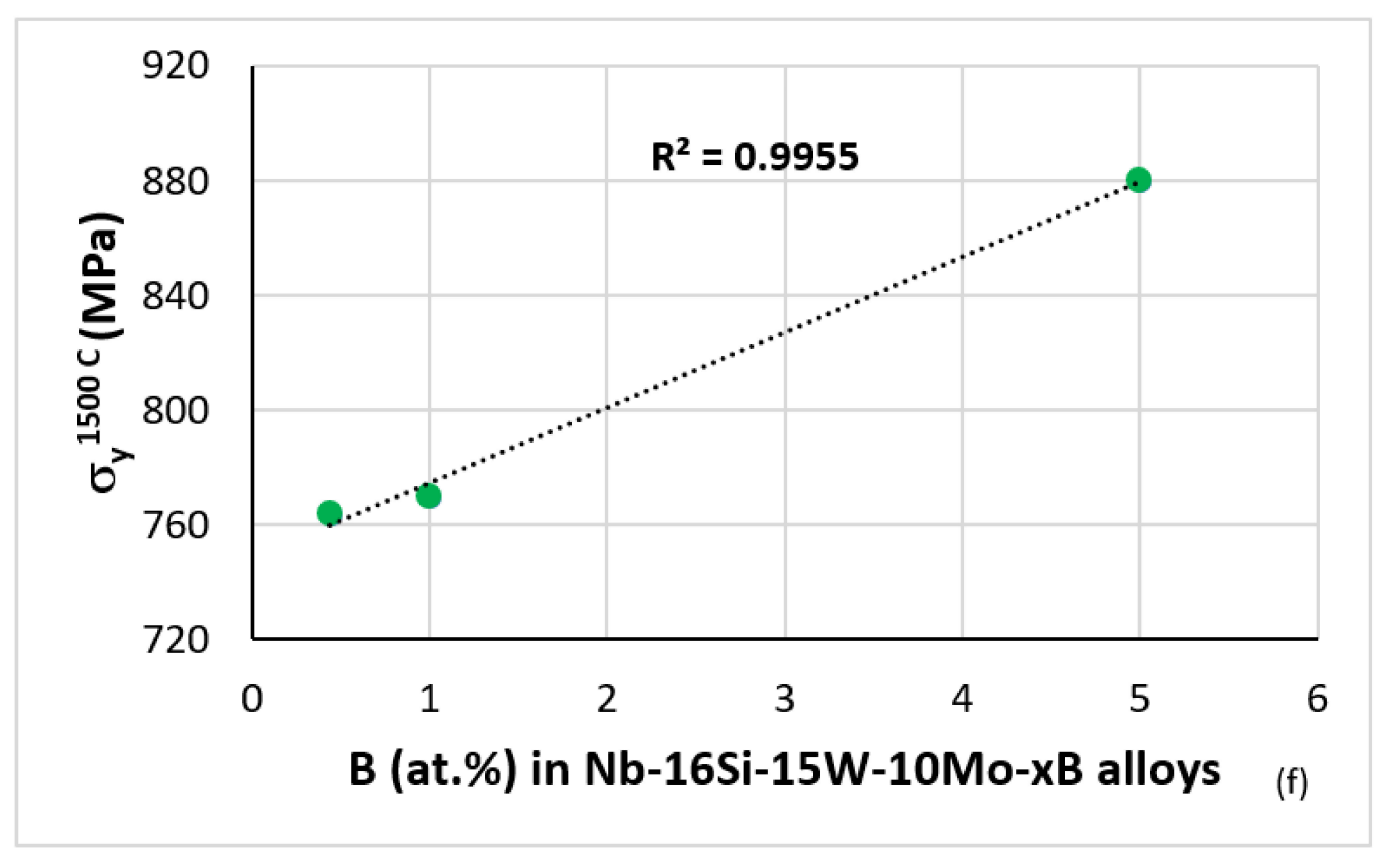
(**a**,**b**) UTS at 1200 °C of solid solution alloys. (**a**) Nb-xMo (x = 0,7.8,10,12,14.5 at.%) versus VEC. (**b**) Nb-xW (x = 8.2, 11.1, 14.2 at.%) versus W content. (**c**) Minimum creep rate at 1200 °C and 140 MPa versus VEC. (**d**) Rupture life at 1200 °C and 140 MPa versus VEC of solid solution Nb-xW alloys (x = 8.1, 11.5, 14.5, 18 at.%) [29,30]. (**e**) Compressive yield stress at 1200 °C of Nb–10W–10Si–*x*B alloys (x = 0, 0.1, 1 and 2) [31]. (**f**) Compressive yield stress at 1500 °C of Nb-16Si-15W-10Mo-xB alloys x = 0.44, 1, 5) [32].

The literature shows a preference for RM = Mo,Nb,Ta,W additions in metallic UHTMs [1,2,3,4,7]. These RMs give strong solid solution strengthening to the bcc solid solution(s) (for example see Figure 7b and [2,3]) influence the type of bcc solid solution that can form, and/or be stable in the microstructure of RM(Nb)ICs, RM(Nb)ICs/RCCAs or RM(Nb)ICs/RHEAs, meaning “normal” or Ti rich or Si free or B free (see [1,3,8,13]) and improve creep [1,3]. According to NICE, the UTS, yield stress and creep of metallic UHTMs improves as the alloy parameter VEC_alloy_ increases [1], which is supported by experimental data [1,3,4]. For example, Figure 7a shows that the UTS of Nb-Mo alloys increased with increasing VEC_alloy_, and Figure 7c,d shows improvement of the creep of Nb-W alloys at 1200 °C and 140 MPa as their parameter VEC_alloy_ increased. The yield stress of Ti-free RM(Nb)ICs also increased with the addition of B, particularly when B was in synergy with Mo and W simultaneously (Figure 7e,f).

**Figure 8:**
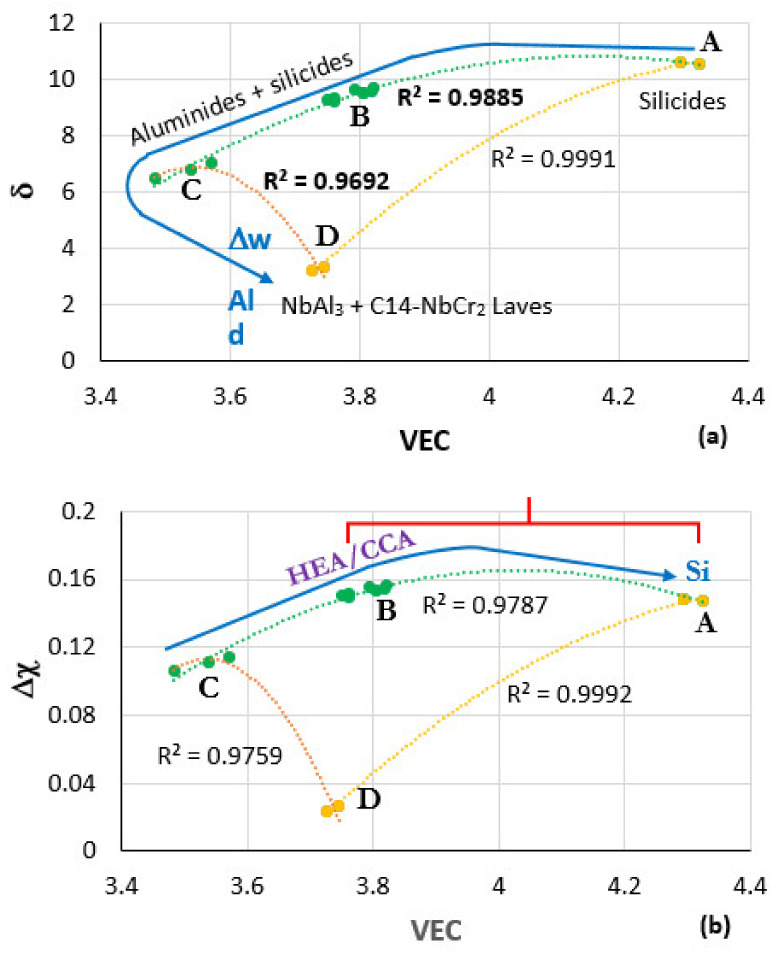
Maps of the parameters δ, Δχ, and VEC with data for the alloys OHC5 [39], MG5, MG6, MG7 [11,22], Zone A of MG7 [11] and OHC3 [10]. In the maps, A corresponds to OHC5, B to MG5, MG6, MG7, C to Zone A of MG7, and D to OHC3. The microstructures in different areas of the maps and the “direction” of increase in Al concentration, mass change per unit area (Δw) and oxide scale thickness (d) are shown in (**a**). The area where alloys could be considered as HEA or CCA and the direction of increase of Si concentration in the map are shown in (**b**). The area where the oxidation rate constants of the BC alloys are in the range of NiAl and Ni-Cr-Al alloys at 1200 °C is shown with the red bracket in (**b**).

Figure 1, Figure 2, Figure 3, Figure 4, Figure 5, Figure 6 and Figure 7 and [1,3,4,7,8,9,14,15] show that the Δχ versus δ and Δχ versus VEC master maps of alloys and their phases can describe the alloying behaviour of metallic UHTMs and their phases and that the parameters δ, Δχ, and VEC correlate with properties of alloys and their phases. This makes it possible to design and/or select new alloys for alloy development research by using the master maps and relationships between the said parameters and properties within the framework of the alloy design methodology NICE [1,3,4].

## 5. Efficacy of Maps of Alloys and Their Phases

The master maps of alloys (Figure 1) and their phases (Figure 3a) are an indispensable part of NICE. What is the usefulness of these maps for the metallurgist who develops new metallic UHTMs? Can they assist him/her to select new alloys worthy of research and development work? Can they point to new experimental research to develop alloys with a balance of creep properties with remarkable (for metallic UHTMs) oxidation behaviour?

Possible routes to alloy design by using NICE were discussed in [1,3,4] and examples of the application of NICE to design/select new alloys were given in [11,13,20,21,22,27]. The alloying elements in RM(Nb)ICs include transition metals, refractory metals, simple metal, and metalloid elements [7]. Electropositive and electronegative elements are present in RM(Nb)ICs [7], in the bcc Nb solid solution(s) [8], in the Nb_5_Si_3_ silicide where they substitute Nb or Si [9], in the C14-NbCr_2_ Laves phase where they substitute Nb or Cr [14], and in the A15-Nb_3_X (X = Al,Ge,Si,Sn) where they substitute Nb [14]. The aforementioned phases are contaminated with oxygen during oxidation, the bcc solid solution more severely, and the severity and “penetration” of contamination (meaning how deeply below the surface contamination occurs), depend on chemical composition and the size and spatial distribution of phases [1,3,4]. In the oxidation of RM(Nb)ICs, there is interdependence of solubility and diffusivity of oxygen, oxidation and diffusion of Nb and solute elements, and chemistry and structure of the oxides, on atomic size, electronegativity, and VEC [1]. The relationship between solute atomic size and diffusivity in Nb was discussed in [8]. The parameter VEC that gives the number of valence electrons per atom filled into the valence band is key to determining the Fermi level in the valence band. Oxygen dissolved interstitially in octahedral holes in bcc Nb will cause electron redistribution and thus the Fermi level will change. The atomic sizes of elements that participate in the oxide scales formed on RM(Nb)ICs are important because the oxide structure consists of blocks of the ReO_3_ type within networks of octahedral MO_6_ groups (M = metal, e.g., Nb, Ta, Ti) [1].

The deformation of bcc metals is controlled by dislocation mobility (intrinsic resistance to dislocation motion, interaction of dislocations with conduction electrons and phonos), i.e., by the motion of kinks. The atomic configuration at a kink is very different than it is in the normal crystal structure. Kink mobility is directly related to electronic structure. Dislocation mobility depends strongly on chemical bonding. In simple metals the bonding is much delocalised. In covalently bonded crystals, the bonding is highly localised. In the transition metals, the electrons that contribute most to the cohesion are localised in spd-hybrid bonds. Correlations exist between the cohesive properties and electronic structure band. Covalently bonded crystals possess intrinsic plastic resistance. The motion of dislocations is limited by the motion of their cores, and the core motion is limited by the motion of kinks along the cores. The bonding in bcc metals gives the screw dislocations a non-planar core structure, the cores are non-degenerate and spatially spread. The spreading varies locally depending on local atomic composition. The location of the Fermi level is indicative of phase stability. For intermetallics a pseudo-gap in the density of states is observed close to the Fermi level owing to combined effects of charge transfer and hybridization. When there is large electronegativity difference between the elements, the redistribution of electrons changes the shape of the band. The changes of the Fermi level resulting from alloying will affect the stability of phases and their properties, both of which are important in creep [1].

In the microstructures of RM(Nb)ICs, bcc Nb solid solution(s) co-exist with covalently bonded compounds (silicides and other intermetallics) [1,3,4,8,9,14,15]. The creep properties of RM(Nb)ICs are key to their application at high homologous temperatures where diffusion is important. Diffusivities in the bcc solid solution and Nb_5_Si_5_ silicide will depend on chemical composition and in the case of the silicide will also depend on crystal structure. The latter changes as solutes partition to the silicide [1,9]. The link between the creep properties of RM(Nb)ICs and their parameters δ (related to atomic size), Δχ (related to electronegativity) and VEC, which relate with the electronic structure of the alloys, is attributed primarily to the covalently bonded intermetallics that are present in their microstructures and to the increase of the covalence of the Nb_ss_ with alloying [1].

When the experimental steady-state creep rate έ of RM(Nb)ICs is considered, relationships are found to exist between έ and each of the aforementioned parameters [1,3,4]. How much each of the parameters δ, Δχ, and VEC contributes to έ is not known [1]. Similarly, when the experimental mass change of RM(Nb)ICs after isothermal oxidation at a particular temperature is considered, relationships are found to exist between mass change per unit area and each of the aforementioned parameters [1,18,21]. However, how much each parameter contributes to mass change in isothermal oxidation is not known.

The location of B-containing RM(Nb)ICs in the right hand corner of the triangles of Figure 2a,c,d and in the left hand side of Figure 2b, respectively is because of the high value of the parameter δ, which is typical for B-containing RM(Nb)ICs and RM(Nb)ICs/RCCAs, and the effect of alloying with B on the creep of Nb_5_Si_3_ (Figures 2c and 12 in [9]) and the shift of Nb_5_Si_3_ in the Δχ versus VEC map when Si and Nb is substituted with B and other simple metal or metalloid elements and Nb with other transition or refractory metals (see Figure 6 in [4], and Figures 5 and 7–10 in [9]).

In NICE, the aforementioned parameters work in synergy and guide the alloy designer to design and select alloys for development. In [4] I wrote “This they do … like the keys of the piano, each playing a single note, but combine them in NICE as you would combine piano keys, and you can create ‘melodies of infinite variety’. Put all these parameters together in NICE, and you have ‘the great symphony of RM(Nb)ICs, HEAs, RHEAs, RCCAs’”. In the same paper [4], I used the “metaphor of the rope” to account for the capabilities (“strength”) of NICE. I wrote “A rope is made of many filaments, but not a single filament goes through the rope’s entire length. It is the way the filaments overlap and their properties that give the rope its strength. Now think of NICE as a rope and the aforementioned parameters its filaments. The capability of NICE to predict … isothermal oxidation behaviour in the pest oxidation regime and at high temperatures, and steady-state creep rates for different temperatures and stresses, and its capacity to calculate compositions of alloys and their phases is found in (results from) the overlap of the aforementioned parameters.”

The master map of metallic UHTMs with Nb and Si addition (Figure 1) is “busy” with data for a wide range of metallic UHTMs and it is difficult to identify in this map the space for new alloys that are worthy of research and development, for example alloys that could offer a balance of properties. Nevertheless, the master map provides another route to designing/selecting alloys. Indeed, by using the relationships in NICE between parameters and creep rate for specific conditions of temperature and stress, Figure 2b can separate the creep-resistant RM(Nb)ICs and RM(Nb)ICs/RCCAs from the oxidation-resistant RM(Nb)ICs and RM(Nb)ICs/RCCAs, some of which have the same alloying additions that promote creep resistance plus other elements that improve oxidation resistance, e.g., the alloys JZ4, JZ5, NT1.1, TT6, TT7, and TT8 (see Appendix A for the nominal compositions).

In Figure 2b the creep-resistant alloys are “stuck” between oxidation-resistant alloys. The latter include alloys that do not pest (Figure 2c) and alloys that do not pest and whose scales do not spall off at low and high temperatures (Figure 2d). In NICE, there are correlations between the parameters δ, Δχ, and VEC of alloys and phases and between the parameters δ, Δχ, and VEC and the properties of alloys and their phases, as discussed in the previous section. On the right-hand side of the creep-resistant alloys in Figure 2b, three areas are shown. One is a rectangle delineated with black lines, and the other two are the light grey and darker grey rectangles. Alloys in the latter two rectangles are B-free and Ti-containing metallic UHTMs with Nb and Si addition and with different vol.% Nb_ss_ and have the potential to offer a balance of properties. The corresponding areas in the master map in Figure 1 are demarcated with 8.34 < δ < 8.38 and 0.181 < Δχ < 0.206, which is related with the light grey rectangle, and with 8.7 < δ < 9 and 0.185 < Δχ < 0.202, which is allied with the darker grey rectangle. In the δ, Δχ, and VEC alloy spaces, the corresponding ranges of the parameter VEC, respectively are 4.615 < VEC < 4.781, and 4.591 < VEC < 4.781. Alloys in the black rectangle in Figure 2b are B-free metallic UHTMs with Nb and Si addition with creep closer to the creep goal. The corresponding area in the master map in Figure 1 is defined with 8.15 < δ < 8.92, in which the concentrations of Mo, Si, and Ti and Al, Cr, and W in the alloy, respectively, increase and decrease with increasing δ, and with 0.24 < Δχ < 0.31, in which the concentrations of Mo, Si, and W, and Ti in the alloy, respectively, increase and decrease with increasing Δχ. In the δ, Δχ, and VEC alloy spaces, the corresponding range of the parameter VEC is 4.776 < VEC < 4.838.

## 6. Maps for Bond Coat Alloys

In NICE, the parameter VEC_alloy_ decreases from high values for the creep goal to low values for the oxidation goal, and the opposite is the case for the parameter δ_alloy_. The aforementioned trends have been confirmed for the oxidation of RM(Nb)ICs and RM(Nb)ICs/RCCAs. For example see [18,33,34] (notice that the alloying with Al, Hf, or Ti of single-phase bcc solid solution RCCAs, studied by Senkov et al. [2], improved their oxidation resistance, as would be expected from the reduction of VEC, see Figure 3a). According to NICE, the parameter Δχ is important for creep rather than oxidation. In consequence, NICE (a) “warns” the metallurgist who designs new metallic UHTMs that in order to suppress pesting and improve oxidation resistance at high temperatures, the parameter VEC_alloy_ should be low and (b) “cautions” him/her that it is unlikely that metallic UHTMs could be designed that meet both the oxidation and creep goals simultaneously (fracture toughness is not considered in NICE). Regarding (b), the same conclusion was reached by Bewlay et. al. [35] for RM(Nb)ICs. For that reason, metallic UHTMs that meet some of the property target(s) and/or are close to other target(s) (i.e., alloys that offer a balance of properties to be considered candidate materials for use in aero engines) will require environmental coatings (EC) [36]. Accordingly, new alloys designed with the help of the alloy master map, as discussed in the previous section, will require ECs.

Most likely, an EC for use with metallic UHTMs will consist of metallic bond coat (BC)/thermally grown oxide (TGO)/top coat (TC) [37]; in other words, the EC could be of the BC/TGO/TC type [38]. The EC must also provide protection against contamination by CMAS (CaO-MgO-Al_2_O_3_-SiO_2_). A prerequisite for the substrate alloy is to have adequate oxidation allied with the oxidation goal, and for the BC to be compatible with the substrate. It is desirable for the BC to form an adhering αAl_2_O_3_ scale as the TGO. Furthermore, the BC could consist of layers of different materials because it must also shield the substrate from interstitial contamination [37,38]. RM(Nb)ICs and RM(Nb)ICs/RCCAs cannot form αAl_2_O_3_ scales [1,3]. The same seems to be the case for the other RCCAs studied to date [2].

Maps of the parameters δ, Δχ, and VEC can assist the alloy designer with his/her task to select suitable BC alloys. In this section, ranges of values of the aforementioned parameters for BC alloys will be indicated with the help of the Figure 8. Figure 8 shows the δ versus VEC and Δχ versus VEC maps of BC Nb-Ti-Si-Al-Cr-Hf alloys that can form αAl_2_O_3_ [10,11,22] and are compatible with Nb-Mo-W-Ti-Cr-Hf-Al-Ge-Si-Sn metallic UHTM substrates (e.g., see [20,21]). The data for the alloys OHC3, OHC5, MG5, MG6, and MG7 (see Appendix A for the nominal compositions) was used to construct these maps. The capital letters A to D indicate where these alloys belong in the maps. A is for the alloy OHC5 [39], B is for MG5, MG6, and MG7 [22], C is for the Zone A of MG7 [11], and D is for the alloy OHC3 [10]. Note that the αAl_2_O_3_ scale forming HEA Nb_1.3_Si_2.4_Ti_2.4_Al_3.5_Hf_0.4_ (alloy MG7), owing to the combined effects of macrosegregation and solidification at high cooling rates, separated to an Al rich “normal” intermetallic alloy (Zone A, [11,22]), a less Al rich normal Al intermetallic alloy and the HEA alloy in the bulk of the arc melted button (see Section 3).

The concentration of Al increases from the alloy OHC5 to OHC3, and the concentration of Si increases from Zone A of the alloy MG7 to the alloy OHC5, as shown with the blue arrows in the Figure 8a,b, respectively, for Al and Si. The increase of mass change per unit area (∆w) and thickness (d) of scale at 1200 °C from the alloy OHC5 to OHC3 is also shown with the blue arrow in Figure 8a. Different intermetallics form in the alloys in the different areas of the maps (see Figure 8a).

The nominal compositions of the BC alloys in the area near B meet the standard definition of HEAs or CCAs (see Figure 8b). The parabolic rate constant of typical alumina scales formed on Ni-Cr-Al alloys in air at 1200 °C is about 5.6 × 10^−12^ g^2^/cm^4^s [40]. The red bracket in Figure 8b shows the part of the map where the alumina-forming BC alloys with aforementioned alloying elements form alumina scales with rate constants in the range of the kp values of NiAl and Ni-Cr-Al alloys.

Zone A formation was observed in the alloy MG7 and layered microstructures formed in the alloys MG7 and OHC5 [10,11]. Common in all these alloys was the simultaneous presence of Al, Si, and Ti and solute macrosegregation. Note that in the alloy OHC3, which did not contain Si and Ti, a Zone A did not form even though the alloy was Al-rich. Considering the maps in the Figure 8, if Zone A formation in the BC alloy were to be required, the alloy should have δ > 6, VEC > 3.75 and Δχ > 0.1. BC alloys with Al, Cr, Hf, Nb, Si, and Ti-alloying elements compatible with metallic UHTM substrates with Nb and Si addition could be designed/selected to have the parameters δ, VEC, and Δχ in the as-manufactured condition and after exposure to high temperature, respectively, in the ranges 6 < δ < 10.5, 3.75 < VEC < 4.5 and 0.1 < Δχ < 0.16.

## 7. Conclusions

Metallic ultrahigh-temperature materials (UHTMs) (i.e., RM(Nb)ICs, RM(Nb)ICs/RCCAs, PM(Nb)ICs/RHEAs, and RHEAs and RCCAs with Nb and Si addition) and the alloying behaviour and properties of alloys and their phases were considered by using maps of the parameters δ, Δχ, and VEC, which are indispensable in the alloy-design methodology NICE. What connects and what differentiates the material groups was discussed by using parameter maps. Attention was drawn to the fact that high-entropy or complex concentrated intermetallics, namely 5-3 silicides, C14 Laves and A15 compounds, and bcc solid solutions and eutectics can form in metallic UHTMs where such phases co-exist with conventional phases. The practicality of maps for the design/selection of substrate alloys with different properties was deliberated. The need for environmental coatings for metallic UHTMs was considered, and the design of bond coat alloys was discussed by using relevant maps.

## Figures and Tables

**Table 1:** Actual chemical composition (at.%) of high-entropy or complex concentrated phases, namely bcc solid solutions (Nb_ss_), tetragonal Nb_5_Si_3_ silicides, eutectics with Nb_ss_ and tetragonal Nb_5_Si_3_, A15-Nb_3_X (X = Al,Ge,Si,Sn) compounds, and C14-NbCr_2_ Laves phase, that can form in RM(Nb)ICs and RM(Nb)ICs/RCCAs, and the metallic UHTM. For nominal compositions of alloys see Appendix A. (AC = as cast, HT = heat treated).

Phase	Element													UHTM and Condition
	Al	B	Cr	Ge	Hf	Mo	Nb	Si	Sn	Ta	Ti	V	W	
Nb_ss_	5.6	-	6.8	-	5.3	-	36.6	1.6	2.7	-	32.8	8.7	-	NV1-AC
Nb_ss_	6.7	-	10.9	-	3.7	-	35.9	1.7	4.7	-	36.4	-	-	EZ8-AC
Nb_ss_	7.2	-	29.4	1.5	-	-	23.3	5.3	2.7	-	30.6	-	-	OHS1-AC
Nb_ss_	8.6	-	19.1	1.2	2.3	-	26.4	2.4	3.5	3.6	31.4	-	1.5	JZ3-AC
Nb_ss_	5.6	-	12.2	0.5	0.3	-	40.6	2.9	2.5	10.4	14.1	-	10.9	JZ3-AC
Nb_ss_	1.5	-	7.1	-	-	-	30	2.1	0.4	15.1	3.9	-	39.8	JZ3^+^-HT
Nb_ss_	5.3	-	17.7	1.6	0.3	23.4	21.2	-	1.2	-	11.3	-	18	JZ5-HT
Nb_ss_	7	0.7	15.7	-	-	-	35.6	1.3	-	-	39.7	-	-	TT4-AC
Nb_ss_	7.1	-	15.9	-	-	-	31.4	1.4	-	3.7	40.2	-	-	TT5-AC
Nb_ss_	6.8	1.1	15.6	-	-	2.4	34.5	1.2	-	-	38.4	-	-	TT8-AC
Nb_5_Si_3_	3.2	-	1.4	-	-	-	38.3	34.9	-	4.5	17.7	-	-	KZ6-AC
Nb_5_Si_3_	3.5	-	2.2	-	-	1.5	35.7	30.6	-	-	26.5	-	-	JG2-HT
Nb_5_Si_3_	3.7	-	0.4	-	9.6	-	29.3	34.1	0.4	-	20.7	1.8	-	NV1-AC
Nb_5_Si_3_	4.1	-	1.8	-	10.3	-	25.7	32	1.4	-	24.6	-	-	EZ8-AC
Nb_5_Si_3_	5.2	-	1.9	6.7	-	-	38.8	20.6	3.8	-	23	-	-	OHS1-AC
Nb_5_Si_3_	4.6	-	3.5	6.9	1.6	-	37.3	23.1	2	4.5	15.9	-	0.6	JZ3-AC
Nb_5_Si_3_	4.6	-	3	5.6	0.9	-	39.2	23.8	4.2	4.9	13	-	0.8	JZ3^+^-HT
Nb_5_Si_3_	5.2	-	4	7.2	3.2	3.8	27.4	24.1	1.4	-	23.7	-	-	JZ5-HT
Nb_5_Si_3_	4	4.4	1.5	-	-	-	32.5	28.3	-	-	29.3	-	-	TT4-AC
Nb_5_Si_3_	3.9	5.4	2.2	-	-	-	25.4	27.7	-	2.6	32.8	-	-	TT5-AC
Nb_5_Si_3_	4.3	5.8	1.4	-	-	-	33	27.4	0.8	-	27.1	-	-	TT6-AC
Nb_5_Si_3_	3.3	8	0.9	-	-	0.5	30.7	26.4	-	-	30.2	-	-	TT8-AC
Nb_5_Si_3_	4.8	-	2.7	7.8	-	-	37.6	21	-	-	26.1	-	-	ZF6-HT
Nb_5_Si_3_	3.2	-	2.3	6.7	4.8	-	37	24.8	-	-	21.2	-	-	ZF9-HT
Nb_5_Si_3_	3.7	-	2.6	-	-	-	35.2	29.2	1.8	-	27.5	-	-	ZX8-HT
**Phase**	**Element**													**UHTM and condition**
	**Al**	**B**	**Cr**	**Ge**	**Hf**	**Mo**	**Nb**	**Si**	**Sn**	**Ta**	**Ti**	**V**	**W**	
eutectic	4.6	-	1.3	-	7.7	-	39.6	18.2	1.6	-	23.4	3.6	-	NV1-AC
eutectic	5.4	-	-	-	7.3	-	38.8	13.4	4.4	-	30.6	-	-	EZ5-AC
A15	7.2	-	16.5	4.3	2.6	-	26.8	9.8	3	3.2	25.6	-	1	JZ3-AC
A15	6.4	-	8.7	0.9	0.5	-	39.9	2.8	12.6	4.9	19.3	-	4	JZ3^+^-AC
A15	9.4	-	12	0.9	-	14.4	30	1.1	12.5	-	15.1	-	4.6	JZ4-AC
A15	7.1	-	9.5	1.2	-	14.3	39.4	2	10.3	-	10.6	-	5.6	JZ4-HT
A15	7.1	-	7.4	2.1	0.5	16.2	30.1	2.1	10.3	-	19.4	-	4.8	JZ5-AC
A15	10.5	-	14.1	1.4	0.4	15.5	19.1	0.8	11.6	-	22.7	-	3.9	JZ5-AC
A15	7.3	-	10.8	1.8	-	16.3	30.1	2.4	9.2	-	17.4	-	4.7	JZ5-HT
Laves	-	-	38.4	-	-	-	20.8	10.7	1.8	-	28.3	-	-	ZX4-AC
Laves	2.7	-	38.8	-	-	-	27	9.6	1.1	-	20.8	-	-	ZX8-AC
Laves	3.5	-	41.6	-	5.5	-	22.7	8.3	-	-	18.4	-	-	JG4-AC

## Data Availability

No new data were created in this study.

## Appendix A

**Table A1:** Nominal compositions (at.%) of reference alloys used in this work.

Alloy	Element														
	Nb	Ti	Si	Al	Hf	Cr	Mo	Ta	V	W	Zr	B	Ge	Sn	Ref.
EZ5	43	24	18	5	5	-	-	-	-	-	-	-	-	5	[41]
EZ8	38	24	18	5	5	5	-	-	-	-	-	-	-	5	[41]
JG2	43	24	18	5	-	5	5	-	-	-	-	-	-	-	[42]
JG4	41	24	18	5	5	5	2	-	-	-	-	-	-	-	[42]
JG6	36	24	18	5	5	5	2	-	-	-	-	-	-	5	[42]
JN1	43	24	18	5	5	5	-	-	-	-	-	-	-	-	[43]
JZ3	40.5	12	18	5	1	5		6	-	2.5	-	-	5	5	[20]
JZ3+	38	12	18	5	1	5	-	6	-	2.5	-	-	5	7.5	[20]
JZ4	43.8	11.5	18	4.7	1	4.5	5	-	-	2	-	-	4.6	4.9	[21]
JZ5	36	21	18	3.7	0.8	4	6.7	-	-	1.2	-	-	4.2	4.4	[21]
KZ6	42	24	18	5	-	5	-	6	-	-	-	-	-	-	[26]
MG5	15.1	24.2	26.7	30.6	3.4	-	-	-	-	-	-	-	-	-	[22]
MG6	12.8	22.7	23.2	37.7	3.6	-	-	-	-	-	-	-	-	-	[22]
MG7	13	24	24	35	4	-	-	-	-	-	-	-	-	-	[11]
NT1.1	33	20	19	5	1	5	6	-	-	1	-	-	5	5	[44]
NV1	53	23	5	5	5	2	-	-	5	-	-	-		2	[45]
OHC3	25.5	-	-	66	0.5	8	-	-	-	-	-	-	-	-	[10]
OHC5	25	5	60	5	-	5	-	-	-	-	-	-	-	-	[39]
OHS1	38	24	18	5	-	5	-	-	-	-	-	-	5	5	[18]
PT1	63	10	18	-	1	-	5	-	-	3	-	-	-	-	[46]
PT2	64	10	18	-	-	-	5	-	-	3	-	-	-	-	[46]
PT3	59	10	18	-	5	-	5	-	-	3	-	-	-	-	[46]
PT4	54	10	18	5	5	-	5	-	-	3	-	-	-	-	[46]
TT4	40	24	18	5	-	5	-	-	-	-	-	8	-	-	[12]
TT5	37	24	18	5	-	5	-	6	-	-	-	5	-	-	[13]
TT6	39	24	18	4	-	5	-	-	-	-	-	6	-	4	[13]
TT7	38	24	17	5	5	5	-	-	-	-	-	6	-	-	[13]
TT8	42.5	24	17	3.5	-	5	2	-	-	-	-	6	-	-	[12]
XX1	59	-	16	-	5	-	5	-	-	15	-	-	-	-	[46]
XX2	37	-	18	-	5	-	20	10	-	5	5	-	-	-	[46]
XX3	47	10	18	-	-	-	10	-	-	15	-	-	-	-	[46]
XX4	57	10	18	-	-	-	10	-	-	5	-	-	-	-	[46]
XX5	40	22	18	-	-	-	20	-	-	-	-	-	-	-	[23]
XX6	50	22	18	-	-	-	10	-	-	-	-	-	-	-	[46]
YG4	72	-	18	-	5	-	2	3	-	-	-	-	-	-	[47]
YG5	70	-	20	-	5	-	-	-	-	5	-	-	-	-	[24]
YG6	72	-	20	-	-	-	5	-	-	3	-	-	-	-	[24]
YG8	67	-	20	-	5	-	5	-	-	3	-	-	-	-	[24]
ZF6	43	24	18	5	-	5	-	-	-	-	-	-	5	-	[19]
ZF9	38	24	18	5	5	5	-	-	-	-		-	5	-	[19]
ZX4	48	24	18	-	-	5	-	-	-	-	-	-	-	5	[34]
ZX8	43	24	18	5	-	5	-	-	-	-		-	-	5	[34]

KZ series alloys: Nb-24Ti-18Si, Nb-24Ti-18Si-5Al, Nb-24Ti-18Si-5Cr, Nb-24Ti-18Si-5Al-5Cr, Nb-24Ti-18Si-5Al-5Cr-6Ta [24,25].
